# Acceptability, Feasibility, and Preliminary Effectiveness of a Wellbeing Coordination Program in an Integrated Health and Social Care Hub: A Mixed Methods Study

**DOI:** 10.5334/ijic.8644

**Published:** 2025-02-19

**Authors:** Lingling Chen, Natalie White, Emma Patten, Danielle Barth, Leanne N. Constable, Teresa Hall, Ashraful Kabir, Harriet Hiscock, Sarah Loveday

**Affiliations:** 1 Health Services, Murdoch Children’s Research Institute, Melbourne, Victoria, Australia; 2 Centre for Community Child Health, The Royal Children’s Hospital, Melbourne, Victoria, Australia; 3 IPC Health, Melbourne, Victoria, Australia; 4 Strategy Planning Improvement and Innovation, Children’s Health Queensland Hospital and Health Service, Brisbane, Queensland, Australia; 5 Department of Paediatrics, University of Melbourne, Parkville, Victoria, Australia

**Keywords:** integrated care, care navigation, social prescribing, childhood adversity, mixed methods

## Abstract

**Introduction:**

Families experiencing adversity often have complex needs and face barriers to accessing health and social care. This study evaluated the acceptability, feasibility and preliminary effectiveness of a Wellbeing Coordination (WBC) program to improve access to services. The program combined care navigation and social prescribing within an integrated health and social care Child and Family Hub.

**Methods:**

Mixed-methods design, with data collected through surveys and interviews from: 1) caregivers who participated in the WBC program (*n* = 11) and those who did not (*n* = 18), and 2) practitioners working in the Hub (*n* = 21).

**Results:**

Caregivers and practitioners found the WBC program acceptable and mostly feasible, demonstrating the potential to alleviate caregivers’ loneliness and enhance their health, connection to the community, and knowledge and confidence in supporting child and family health and wellbeing.

**Discussion:**

Future WBC program enhancements could include a robust communication strategy to ensure what the program offers is clearly understood by practitioners and caregivers, establishing organisational structures to provide adequate support for the wellbeing coordinator and adopting flexible eligibility criteria.

**Conclusion:**

The WBC program appears acceptable and feasible. Future research should establish program effectiveness with larger and more diverse caregiver samples.

## Introduction

Families with children experiencing adverse childhood adversities (ACEs), including maltreatment, household dysfunction, community dysfunction, and peer dysfunction [1], often have complex needs requiring support and care from multiple services [2]. However, they often face barriers to accessing appropriate health and social support, including the significant personal effort it can take to navigate health and social care systems, fear of judgment by service providers, long wait lists and high out-of-pocket costs of services [3]. Care navigation and social prescribing are two approaches that can help address these barriers by increasing access to necessary supports for families.

Care navigation (or system or patient navigation) refers to an individual or a team engaging in specific activities that facilitate access to health and social services for patients and caregivers, to ensure effective and efficient use of the health care system for patients, caregivers, and practitioners [4]. A scoping review of 34 papers investigating navigation delivery models in primary care found that only two studies focused on children (including unborn) living with adversity [4], however one focused exclusively on navigating health (not social) care services [5], and one on a specific type of adversity (i.e., intimate partner violence) only [6].

Social prescribing is another approach that aims to increase access to care by providing linkages or referrals from health to non-health/community-based supports [7]. As the Social Prescribing Network defines, social prescribing enables ‘healthcare professionals to refer patients to a link worker, to co-design a non-clinical social prescription to improve their health and wellbeing’ [7]. Social prescriptions can be diverse and can range from financial advice to walking groups [8], with the potential to reduce loneliness and social isolation, and enhance community connection, health and wellbeing [9,10]. Most studies have focused on social prescribing interventions for adults [11,12], while few have explored the model for children and families [13]. In Australia, social prescribing interventions and research are emerging; there is currently a gap in knowledge investigating the impact of social prescribing for children and families facing adversity [14,15,16,17].

Whilst there are similarities between care navigation and social prescribing (i.e., both involve navigators or link workers linking patients and caregivers to services [3,16]), their focus differs. Care navigation addresses the complexities of navigating the broad and fragmented care system, whereas social prescribing supports patients through non-medical prescriptions. This study aimed to evaluate a Wellbeing Coordination (WBC) program that combined care navigation and social prescribing for families with children aged 0–12 years experiencing adversity. The WBC program was nested within an integrated health and social care Child and Family Hub (hereafter referred to as the Hub) codesigned with families, local community members, and health, social, and family service practitioners, and implemented in 2020–2022 in Wyndham Vale, Victoria, Australia. The purpose of the Hub was to improve children’s mental health by earlier detection and response to family adversity [2].

The overarching evaluation question for the WBC program was ‘What is the feasibility, acceptability, and potential effectiveness of the WBC program?’ Guided by a framework for feasibility studies [18], we evaluated the program as follows: 1) Reach: Did the program reach its intended audience? 2) Fidelity: Was the program implemented as intended? 3) Acceptability: Was the program acceptable to those delivering and receiving it? 4) Feasibility: Was it possible to deliver the program with the resources allocated? and 5) Preliminary effectiveness: Did the program reduce caregiver loneliness, and improve their health, social connection, and knowledge and confidence in managing their family’s health and wellbeing?

## Methods

Using a mixed-methods design, this study collected routine monitoring data (quantitative) and participant data (quantitative and qualitative), to provide a comprehensive evaluation of the program. The Royal Children’s Hospital Human Research Ethics Committee (HREC 62866) approved the study, and participants gave informed consent before taking part.

### Study Setting

This study was conducted as a nested component of the implementation and evaluation of the Hub [19] within a community health centre at IPC Health Wyndham Vale. Wyndham is a culturally diverse region within Greater Metropolitan Melbourne, characterised by population risk factors for family adversity, such as elevated levels of unemployment, housing stress, and social isolation compared to greater Melbourne [20,21].

### The WBC Program

A wellbeing coordinator with a professional background in social work provided support to caregivers in identifying the comprehensive needs of their child and/or family and assisted in connecting them with relevant services and supports in the community. Referral information was received by the wellbeing coordinator via self-referral from a caregiver, internal (within the Hub) or external (external to the Hub) practitioners. The inclusion criteria were codesigned by the research team, wellbeing coordinator, Hub practitioners, social prescribing workers and IPC Health’s management. These included: 1) Caregiver-reported social isolation; 2) recent migration; 3) difficulty navigating community services; 4) need help connecting to the National Disability Insurance Scheme (NDIS; providing funding for children with a disability or with developmental delay, to access the services and supports they need); 5) child with a new diagnosis needing additional support; and 6) waiting for services referred by Hub practitioners. Exclusion criteria aimed to prevent service duplication and confusion, including: 1) involvement with Family Services or Child Protection; and 2) active case management services. The WBC program elements are presented in Table 1.

**Table 1:** WBC Program Elements and WBC Role.


WBC PROGRAM ELEMENTS	WBC ROLE

Appointment format	Up to six support appointments for families

Care navigation	Developing wellbeing care plans with goals and service links

Social prescription	Co-designing nonclinical social prescriptions (e.g., financial counselling, playgroups)

Family connection	Monthly Community Connect sessions for casual caregiver-practitioner engagement and service awareness


### Participants

Caregivers receiving services from the Hub included those who participated in the WBC program and those who did not (but participated in the Hub evaluation), along with practitioners working in the Hub.

#### Caregivers Receiving the Hub Services

Caregivers of children aged 0–12 years (including pregnant women) who understood English were invited to participate in the WBC program evaluation.

The wellbeing coordinator approached caregivers at their first appointment or, for a small sample, after joining the program. They were then invited for an interview at the end of their participation. Participants received an AUD$40 gift voucher for interview participation.

#### Practitioners Working in the Hub

Practitioners collaborating with the wellbeing coordinator in the Hub were invited for an interview (*n* = 21) at the end of the 12-month Hub evaluation. These practitioners, from health and social care at IPC Health, received training and attended monthly meetings to enhance their response to childhood adversity during the Hub’s implementation [19].

### Data Collection

Quantitative (i.e., referral information and caregiver surveys) and qualitative data (i.e., interviews with caregivers and practitioners) were collected to evaluate the WBC program.

#### Referral Information

A range of referral process information was collected by a research team member, including the number of referrals received, the number of referrals not accepted, reasons for referral, sources of referral (internal/external/self-referral), the role of referrers, the number of families receiving services from the WBC program, contact attempts to engage families, number of appointments per family, whether a wellbeing plan was developed, and number of referrals made by time of discharge.

#### Intake and Exit Surveys

Caregiver characteristics and social and psychological status were collected at two time points: upon entry into the WBC program (intake survey) and discharge from the program (exit survey). The instruments used in both surveys are presented in Table 2.

**Table 2:** Instruments Used in Intake and Exit Surveys.


VARIABLES	INSTRUMENTS	INTAKE SURVEY	EXIT SURVEY

Caregiver characteristics	Caregiver age, gender, country of birth, primary languages spoken at home, Aboriginal and/or Torres Strait Islander status, refugee status, educational, and household composition.	X	–

Social isolation	Four items from an index of social isolation [22,23]: unmarried/not cohabiting; infrequent contact with family or friends, and no participation in groups. Items were rated yes/no, with higher scores indicating greater isolation.	X	–

Loneliness	The three-item short form of the Revised UCLA loneliness scale [24]: Items were rated on a 3-point scale (1 = hardly, 3 = often), with higher scores indicated greater loneliness.	X	X

Health	Six items from a 10-item Patient-Reported Outcomes Measurement Information System (PROMIS) Global Health [25] assessed caregivers’ general, physical, mental, and social health, and quality of life. Items were rated on a 5-point scale (1 = poor, 5 = excellent), with higher scores indicating better health.	X	X

Confidence to self-manage health problems	Single item: ‘*How confident are you that you can control and manage most of your health problems*?’ [26]. Rating based on a 10-point scale (1 = not at all, 10 = very).	X	X

Knowledge of local services	Study-designed, single item: ‘*Do you feel like you have a good knowledge of services in the local area to support your child and family’s health and wellbeing*?’ Rating based on a 5-point scale (1 = poor, 5 = excellent).	X	X

Connection to the community	Study-designed, single item: ‘*Have you experienced a greater connectedness in your community since taking part in the WBC program*?’ with participants rating ‘yes’, ‘no’, or ‘somewhat’.	-	X

Caregiver enablement	Six items from a modified Parent Enablement Index [27,28] assessed caregivers’ ability to cope with life, understand and support their children, maintain health, feel confident about their health, and help themselves. Rating based on a 3-point scale (0 = same or less or not applicable, 1 = not confident at all, 2 = much better). The total score ranges from 0 to 12. Scores above 4 indicate greater enablement as a result of the service [29].	–	X

Feedback on the WBC program	Six study-designed question: two on goal achievement and program recommendation (yes/no/other) and four on caregivers’ experiences of being listened to, respected, and supported, rated on a 5-point scale (1 = always, 5 = never).	–	X


#### Interviews

Qualitative data were collected (from March to July 2023) using individual semi-structured interviews with caregivers and practitioners, and one group interview with practitioners. As an exploratory study, the interview schedules were developed based on existing literature on care navigation and social prescribing, along with the objectives of the WBC program, focusing on evaluating its acceptability, feasibility, and effectiveness.

**Caregiver interviews**. A semi-structured interview schedule (see Supplemental File 1) asked about caregivers’ attitudes towards the WBC program when they first learnt of the program, and their experiences of taking part in the program at the conclusion of their participation. The schedule was reviewed by a lived experience researcher [LNC] for content validity and to ensure the language was appropriate for families experiencing adversity. For example, the term ‘life challenge’ was used to describe adversity. Interviews were conducted individually (*n* = 20) by LC, averaging 54 minutes (range: 25–113 minutes).

**Practitioner interviews**. Semi-structured interviews (see Supplemental File 1) were used at the 12-month evaluation to explore practitioners’ experiences of the program. Interviews were conducted individually (*n* = 18) or in groups (*n* = 1 group of 3 nurse practitioners) by SL and LNC, averaging 39 minutes (range: 29–70 minutes).

Interviews were undertaken in person at IPC Health, over the phone, or by videoconference according to participant preference. Interviews were audio recorded and verbatim transcribed using OutScribe, an online transcription service. The transcripts were then checked against the audio recording for accuracy.

### Data Analysis

Given the small sample size, we conducted descriptive analyses of the quantitative referral and survey data using SPSS 28. Interview data were analysed using reflexive thematic analysis and followed the six-phase approach as described by Braun and Clarke [30]: 1) familiarisation with the data, 2) generating initial codes, 3) searching for themes, 4) reviewing potential themes, 5) defining and renaming themes, and 6) producing the report. Coding was inductive and reflexive with keywords or phrases assigned a code using NVivo 14. The transcripts of caregivers and practitioners were analysed separately, and all were coded independently by three researchers (SL, AK, and LC), with each transcript dual-coded by two of the researchers. Codes were discussed to ensure clarity of meaning, and new codes developed by each researcher were reviewed and agreed on. Following the coding process, codes were grouped into categories, and then initial themes were constructed. Themes were discussed and generated in an iterative process until overarching themes were developed by SL and LC through discussion with the broader research team.

### Positionality and Reflexivity

Two members of the research team are practising paediatricians (SL and HH). Other members included a senior research officer (NW), a lived experience researcher (LNC), two researchers in public health (AK and TH), a psychology researcher with expertise in qualitative research (LC), a practitioner (EP) and senior manager in community health (DB). Reflexive notes taken throughout each interview allowed for reflection and interpretation. Credibility was achieved through peer checking, regular debriefing in the team and triangulation of reflexive notes and transcripts.

## Results

### Participant Sociodemographic Characteristics

The demographic characteristics of the three participant groups are summarised in Tables 3 and 4, including caregivers who did not receive any services from the WBC program (*n* = 18), caregivers who participated in the WBC program (*n* = 11), and practitioners working in the Hub (including the wellbeing coordinator; *n* = 21).

**Table 3:** Sociodemographic Characteristics of Caregivers (n = 29).


PARTICIPANT CHARACTERISTICS	n (%)		

	CAREGIVERS NOT PARTICIPATING IN THE WBC PROGRAM BUT COMPLETING INTERVIEWS (*n* = 18)	CAREGIVERS PARTICIPATING IN THE WBC PROGRAM AND COMPLETING SURVEYS (*n* = 9)	CAREGIVERS PARTICIPATING IN THE WBC PROGRAM AND COMPLETING INTERVIEWS (*n* = 2)

**Age M (SD, Range)**	38 (8.01, 25–59)	38.78 (7.82, 30–53)	32 (2.12, 30–33)

**Gender**			

Female	13 (72.2)	8 (88.9)	2 (100)

**Country of birth**			

Australia	9 (50)	5 (55.6)	1 (50)

Asian countries	7 (38.9)	2 (22.2)	0

African countries	2 (11.1)	2 (22.2)	1 (50)

**Primary language spoken**			

English	9 (50)	6 (66.7)	2 (100)

**Aboriginal and/or Torres Islanders**			

Yes	1 (11.1)	0	0

**Highest level of schooling**			

Under Year 12 (final year of schooling)	0	5 (55.5)	1 (50)

Year 12 or equivalent	5 (27.8)	2 (22.2)	1 (50)

Trade or other certificate-level qualification	3 (16.7)	1 (11.1)	0

Bachelor’s degree	5 (27.8)	0	0

Postgraduate qualification	5 (27.8)	0	0

Missing	0	1 (11.1)	0

**Number of children in the household**			

1	4 (22.2)	2 (22.2)	0

2	7 (38.9)	5 (55.6)	2 (100)

3	6 (33.3)	1 (11.1)	0

4 or more	1 (5.6)	1 (11.1)	0

**Social isolation index**			

Unmarried/not cohabitating	N/A	6 (66.7)	N/A

Less than monthly contact with other family members	N/A	1 (11.1)	N/A

Less than monthly contact with friends	N/A	2 (22.2)	N/A

No participation in organisations (e.g., social clubs, residents’ groups)	N/A	5 (55.6)	N/A


**Table 4:** Demographics of Practitioners (n = 21).


PRACTITIONER CHARACTERISTICS	n(%)

**Age**	

18–34 years	4 (19.1)

35–44 years	9 (42.8)

45–54 years	4 (19.0)

55–64 years	4 (19.1)

**Gender**	

Female	18 (85.7)

**Role**	

Paediatrician/paediatric fellow	3 (14.3)

General practitioner	2 (9.5)

Nurse	6 (28.6)

Allied health (speech pathologist, dietician)	2 (9.5)

Social worker	2 (9.5)

Financial counsellor	1 (4.8)

Lawyer	5 (23.8)

**Number of years in role**	

< 2 years	2 (9.5)

3–5 years	6 (28.6)

6–10 years	6 (28.6)

>10 years	7 (33.3)


#### Caregivers not Receiving Services from the WBC Program

Eighteen caregivers who did not receive any services from the WBC program were interviewed, with an average age of 38 years (see Table 3). Most were female, had multiple children, and lived with their partner and children; half were born in Australia and spoke English. The educational level of the participants was evenly distributed.

#### Caregivers Receiving Services from the WBC Program

Eleven caregivers who received services from the WBC program participated, of whom nine completed both the intake and exit surveys, and two were interviewed. Participants engaged with the program for an average duration of six months. Most were in their mid-30s, female, born in Australia, educated to Year 12 or below, spoke English at home and had multiple children in the household (see Table 3).

#### Practitioners Working in the Hub

Twenty-one practitioners (including one wellbeing coordinator) working in the Hub were interviewed. Most were female (85.7%) and had a range of different roles across health and social care, as shown in Table 4.

### Program Reach and Fidelity

As shown in Table 5, the wellbeing coordinator received 56 referrals during the evaluation of the program, of which 48 (86%) were deemed appropriate. The out-of-scope referrals (*n* = 8, 14%) included those with highly complex needs (e.g., involvement in Family Services), and those requiring only other services (e.g., financial counselling or legal support). Half of the referrals (*n* = 29, 52%) were internal, with the remainder self-referrals (*n* = 17, 30%) or from external practitioners (*n* = 10, 18%). Except for caregivers’ self-referral, paediatricians (*n* = 14, 25%) and paediatric fellows (*n* = 10, 18%) were the primary source of referrals.

**Table 5:** Referral Characteristics (n = 56).


REFERRAL CHARACTERISTICS	n(%)

**Referrals received by the wellbeing coordinator**	56 (100)

Appropriate referrals	48 (86)

**Referral received from**	

Internal (in the Hub)	29 (52)

External (outside the Hub)	10 (18)

Self-referral	17 (30)

**Referrer roles**	

Self-referral	17 (30)

Paediatrician	14 (25)

Paediatric fellow	10 (18)

Speech Pathologist	5 (9)

Universal maternal and child health nurse	4 (7)

Dietician	3 (5)

Other	3 (6)

**Reasons for referral** ^a^	

Socially isolated	13 (23)

New to area	16 (29)

Overwhelmed with service navigation	50 (89)

NDIS support	35 (63)

New diagnosis	13 (23)

Waiting for services	25 (45)

**Contact attempts to engage families (Range: 0 –11)**	

0	33 (59)

1–3	13 (23)

4–6	8 (14)

≥10	2 (4)

**Families accepted and booked in to see WBC**	40 (71)

**Families attended the initial appointment (actively engaged)**	36 (64)

**Appointments per family attending initial appointments (Range: 1–22)**	

1–6 appointments	26 (72)

7–12 appointments	7 (19)

13–18 appointments	1 (3)

≥19 appointments	2 (6)

**Wellbeing plans developed**	33 (92)

**Referrals generated in wellbeing plans**	

Internal referrals	26 (41)

External referrals	38 (59)


^a^Could be multiple reasons for referral.

Most caregivers were referred to the WBC program given their overwhelming experiences navigating community services and the need to engage with services (*n* = 50, 89%). Two other commonly cited reasons for referrals were the need for support in connecting with the NDIS to access funding for early childhood early intervention (*n* = 35, 63%), and the need for support whilst on wait lists to access care for their children (*n* = 25, 45%).

Of the 56 referrals, the wellbeing coordinator made more than three attempts to reach and engage 10 (18%) families, including more than ten attempts with two families. Thirty-six families (64%) were actively engaged in appointments with the wellbeing coordinator, the majority of whom had no more than six appointments (*n* = 26, 72%) and got a personalised wellbeing plan (*n* = 33, 92%). This indicates a deviation from the original design of the program, where a maximum of six appointments and a minimum of one wellbeing plan for each family was expected. Further referrals made by the wellbeing coordinator included 26 (41%) internal referrals to practitioners at the Hub, and 38 (59%) external referrals outside the Hub. The wellbeing coordinator organised eight Community Connect Drop-In Sessions over a one-year period.

### Acceptability and Feasibility

All practitioners engaged in the Hub were interviewed; however, despite our efforts, only two caregivers who had received the WBC program completed an interview. Analysis of the interview transcripts from caregivers and practitioners revealed important themes about the acceptability and feasibility of the WBC program, which are presented using verbatim quotes.

#### Caregivers’ Attitudes Towards and Experience of the WBC Program

Caregivers’ attitudes towards and/or experiences of the WCB program were generally positive. This is demonstrated by the following four themes: 1) A ‘valuable’ program to know about, 2) Reduced burden of linking to services, 3) Emotional support brings hope, and 4) Positive impacts on wellbeing (refer to Supplemental File 2 for more representative quotes for each theme).

**A ‘valuable’ program to know about**. Many caregivers who did not take part in the WBC program reported not being aware of the program, while a few were aware of it from emails, posters, and practitioners. However, those aware of this program lacked opportunities for in-depth understanding due to reasons including not having the capacity to delve into the program, geographical distance hindering participation in Community Connect Drop-In Sessions, and lack of immediate need. After learning about the WBC program from the interviewer, some caregivers were keen to ‘know in more detail what that wellbeing program is and how they can help’ (Caregiver, C12). Others, while not currently needing the program, recognised its importance for potential future support for their families.

Caregivers stated that their main expectation of the WBC program was to have a single point of contact to navigate the fragmented service system because life challenges can be numerous and occur together. Other expectations include gaining emotional support to alleviate feelings of isolation, and linking with community groups (e.g., groups for parents of children with additional needs), as demonstrated by C17, ‘you won’t feel you’re alone. There’s somebody there that you can speak to, or there’s somebody there that would help you’.

**Reduced burden of linking to services**. Caregivers who engaged in the WBC program indicated a holistic service experience, reducing their burden of linking in to services and obtaining a service. This holistic experience started with the wellbeing coordinator identifying family needs through specific questions about family adversities, e.g., parenting, intimate relationships, and children’s needs.

The main support caregivers received from the WBC program was a combination of care navigation and social prescribing. The services that caregivers were linked to included but were not limited to NDIS support coordination, financial counselling, legal support, family support workers, childcare services, and child activities (e.g., sports).

Caregivers reported that they valued the coordination between the wellbeing coordinator and the services they were referred to. The wellbeing coordinator regularly contacted families to monitor referral progress, alleviating the burden caregivers would normally face in navigating services independently.

**Emotional support brings hope**. Compared to other health professionals, caregivers noted the wellbeing coordinator had more time to work in depth, which fostered a trusting relationship. Throughout their interactions with the wellbeing coordinator, caregivers felt listened to, understood, and not judged. This trusting relationship was strengthened further by the wellbeing coordinator’s lived experience of being a mother. One participant reflected, ‘We get along great because she’s a mum too. […] We have that rapport. We can talk about it. [….] She can confirm to me that stuff is normal’ (C19). Caregivers highlighted that the trust and emotional support from the wellbeing coordinator brought them hope, as C20 described, ‘She has patience, and the way she talked to me and the way she encouraged me. […] She’s very supportive. It makes me feel like there’s hope’.

**Positive impacts on wellbeing**. As a result of the support provided by the WBC program, including care navigation, social prescribing and emotional support, caregivers reported reduced stress and improved mental health outcomes. For example, C19 reflected, “I became way more calm [*sic*], not walking on eggshells all the time, not worried about if I’m going to bump into their dad or anything like that’ (C19). They also observed positive improvement in their children’s development, with one participant contributing her children’s progress to a less stressful home environment because of the experience in this service.

#### Practitioners’ Experiences of the WBC Program

In general, practitioners experienced the WBC program as the ‘backbone’ (Practitioner, P20) of the Hub. The ‘backbone’ function of the WBC program involves: 1) filling the ‘cracks’ between families’ needs and the fragmented care system, as well as between practitioners’ intentions to meet families’ needs and their limited capacity to deliver; and 2) acting as the central connector. The other two themes indicate areas for program enhancement: 3) Need for a team of wellbeing coordinators, and 4) Redefining the program scope. More representative quotes for each theme can be found in Supplemental File 2.

**Filling the ‘cracks’**. Practitioners believed that the WBC program can be helpful by providing a single point of support for families who ‘fall between the cracks’ (P15) or who have complex needs and require additional support. Practitioners also highlighted the value of emotional support when helping families navigate the care system by making them ‘feel more cared for’ (P21) and reaffirming their efforts in seeking professional support.

The WBC program also filled the cracks between practitioners’ intentions to meet the needs of families and their limited capacity (e.g., having time to assess family needs and having knowledge of referral pathways). For some, the wellbeing coordinator served as a reassuring ‘fallback person’ (P17), enabling them to confidently ask and respond to family adversity.

**The wellbeing coordinator is the central connector**. The wellbeing coordinator played a central role in connecting the Hub practitioners and linking families to services. Practitioners described the wellbeing coordinator as ‘the common denominator to so many things’ (P18) because she was well-equipped with the knowledge of local resources and referral pathways. She adeptly built rapport with families, identified their needs, and linked them to services, being viewed as practitioners’ ‘main link’ (P1), and the ‘bridge’ (P17) or ‘middle point’ (P20) between the practitioners. Moreover, for some practitioners, connecting with the wellbeing coordinator brought them a sense of belonging to the Hub community, as stated by P22, ‘Whenever I’ve been here in the community […] like have a chat with her to see how things are going. I guess she is […] like a really accessible way for me to … connect to the Hub.’

**Need for a team of wellbeing coordinators**. Practitioners frequently expressed their concerns about having only one wellbeing coordinator working in the Hub and highlighted the necessity for a team of wellbeing coordinators. Some were concerned about overwhelming the wellbeing coordinator by making ‘lots of referrals all the time’ (P17), leading them to hesitate in making referrals and try to deal with issues themselves.

Additionally, practitioners recognised the substantial support often needed by caregivers, emphasising the necessity for warm referrals or active linking to services, rather than merely providing a referral for caregivers to navigate on their own. Similarly, from the wellbeing coordinator’s perspective, families with complex needs often required more support than the six sessions initially designed by the WBC program because ‘you touch base every now and then, they [caregivers] still want you to be in contact in case something happens’ (P9). There was concern that this intensive work may require a team of wellbeing coordinators to meet community demand and avoid wait lists for program access.

Another indication that there would be benefits of establishing a team of wellbeing coordinators was that being ‘a team of one’ (P9) made the wellbeing coordinator feel isolated in the role which negatively impacted her job satisfaction.

**Redefining the program scope**. Practitioners emphasised the need to redefine the scope of the WBC program, including expanding the scope of services and clearly articulating it in communications to practitioners, caregivers and staff within the Hub. Firstly, it was widely recognised by practitioners that many caregivers they saw at the Hub had highly complex needs and required additional support. However, practitioners noted that the ‘narrow’ (P20) scope of the WBC program, in terms of the eligibility criteria for family access, hindered its ability to support these high-need families. This resulted in tension between practitioners’ expectations and the wellbeing coordinator’s ability to receive referrals. For example, the wellbeing coordinator strictly adhered to eligibility criteria, which were set in an attempt to manage demand, excluding families currently involved in Family Services or Child Protection from the WBC program. However, some practitioners commented that ‘this selective amount of people that they provide does not suit your [practitioners’] needs’ (P21), and one estimated that ‘that leaves out […] 80% of the clients’ (P15). Therefore, some suggested ‘open [*sic*] up the criteria’ (P16) to include more families with complex needs.

Another challenge practitioners encountered was the difficulty in understanding the role of the wellbeing coordinator, as explained by P20, ‘It’s not as clear as saying like I’m a doctor, I’m a speech pathologist with very clear [descriptions] … Like people know what you do’. Practitioners, therefore, highlighted the need for a clear description of the services provided by the WBC program, which would facilitate effective collaboration between the wellbeing coordinator and other practitioners and promote ‘family self-refer’ (P1).

### Preliminary Effectiveness

To evaluate the preliminary effectiveness of the WBC program, a comparison was made of the social and psychological outcomes of the caregivers before and after their participation in the program. Compared to the intake survey, upon exiting the program caregivers reported relatively lower levels of loneliness, and higher ratings of general health, quality of life, mental health and social activities and relationships (see Table 6). Notably, the confidence in controlling and managing health increased from an average of 6.13 (SD = 2.75) to 8.11 (SD = 2.09).

**Table 6:** Changes in Caregiver Outcomes from Intake and Exit Surveys (n = 9).


VARIABLES (RANGE OF SCORES)	INTAKE SURVEY M (SD) /N (%)*	EXIT SURVEY M (SD) /N (%)*

**Loneliness (3–9)**	5.13 (1.96)	5.00 (1.77)

**Global health (1–5)**		

General health	2.89 (1.27)	3.22 (1.20)

Physical health	3.22 (1.39)	3.22 (1.09)

Quality of life	3.00 (1.23)	3.33 (1.12)

Mental health	2.67 (1.00)	3.33 (1.23)

Social activities and relationships	2.22 (.83)	3.67 (.87)

Usual activities and roles	3.78 (.67)	3.56 (1.13)

**Confidence in controlling and managing health (1–10)**	6.13 (2.75)	8.11 (2.09)

**Knowledge of services to support child and family’s health and wellbeing (1–5)**	2.78 (1.20)	3.00 (1.00)

**Caregiver enablement (0–12)**	N/A	6.63 (3.85)

Greater connectedness in the community since taking part in the WBC program*		

Yes	N/A	5 (55.6)

No	N/A	2 (22.2)

Somewhat	N/A	2 (22.2)

Achieved the goals set out in the wellbeing plan*		

Yes	N/A	6 (66.7)

No	N/A	1 (11.1)

Other	N/A	2 (22.2)

**Feedback on the wellbeing coordinator**		

Listened carefully (1–5)	N/A	1.00 (0)

Show respect (1–5)	N/A	1.00 (0)

Spend enough time with families (1–5)	N/A	1.00 (0)

Recommend the WBC Program to others*		

Yes	N/A	9 (100)


Caregivers’ reported ability to cope with life, maintain their own health, and understand and deal with their children’s issues improved slightly through the program. Their knowledge of services that support child and family health and wellbeing was also enhanced. The majority achieved their goals set in the wellbeing plan (*n* = 6, 66.7%), and indicated feeling more connected to the community (*n* = 7, 77.8%) as a result of participating in the program. All the participants gave positive feedback on the work of the wellbeing coordinator, reporting that they were listened to carefully, were respected and had enough time to talk to the wellbeing coordinator. All indicated that they would recommend the program to others.

## Discussion

This study aimed to evaluate the acceptability, feasibility, and preliminary effectiveness of the WBC program, a key component of an integrated health and social care Hub. The WBC program was generally implemented as intended, providing care navigation and social prescribing to families with children experiencing adversity. Both caregivers and practitioners assessed the WBC program as acceptable and feasible despite some challenges related to program capacity. Both quantitative and qualitative results indicate that the WBC program was promising in terms of promoting the mental health and wellbeing of caregivers and their children.

The WBC program reached its intended audience (i.e., families with children experiencing adversity). The majority of referrals received were from paediatricians or paediatric fellows and not the broad range of health and social care practitioners that were a part of the Hub. This is not surprising, given that the majority of families referred to the WBC program were facing challenges in accessing NDIS, and their paediatricians were often the ones identifying these needs. Conversely, other Hub practitioners were still gaining confidence and competence in identifying adversity in their clients and, therefore, were less aware of the support needed and less likely to make referrals to the WBC program [31]. To increase referrals to the WBC program, clearer communication to practitioners may be required, so that they understand the services offered by the WBC program, especially given that the role of wellbeing coordinator (a combination of care navigator and link worker for social prescribing) is still in its infancy in health and social care systems [8,16].

Another important source of referrals was self-referral. It was encouraging that one-third of participating families self-referred to the WBC program. However, according to the caregivers and practitioners interviewed, the self-referral rate could be increased by improving the way the WBC program was communicated to caregivers, including a simpler explanation of the services on offer and the inclusion criteria to take part. Previous research conducted in Wyndham identified that caregivers require support with understanding available services and how to access these services [3,32].

In contrast to traditional care navigation models [4], the WBC program combined care navigation (i.e., providing information and linking caregivers to health and social services) and social prescribing (i.e., co-designing nonclinical social prescriptions). Although this created great opportunities for timely referral to social prescription and to services such as legal support and financial counselling, it was found that referrals for care navigation were greater than for social prescribing. This is not surprising as existing evidence has shown that clients with complex needs in primary care experience fragmentation and gaps in service delivery [4].

Caregivers and practitioners interviewed found the WBC program acceptable, highlighting its ‘backbone’ function in the Hub. It provided a single point of contact which enabled holistic care navigation, reducing the caregivers’ burden of linking to health and social care services. As other research has found, addressing barriers to accessing support is crucial because seeking help for adversity can be emotionally taxing and effortful for caregivers especially when navigating complex care systems [3,33]. For practitioners, having the WBC program boosted confidence in identifying family adversity, resonating with findings of other studies that practitioners are more likely to respond to clients’ adversity when confident in accessing appropriate support [34,35]. Additionally, the WBC program instilled hope in caregivers and fostered a sense of belonging among Hub practitioners, which was unexpected but inspiring. This underscores that the role of the wellbeing coordinator was not only a practical resource but also facilitated meaningful social connections for practitioners and caregivers.

Whilst the WBC program was feasible, existing funding sources were not adequate to sustain a role of this nature, resulting in limited capacity to support families. This resulted in tensions between practitioners and the wellbeing coordinator, and a risk of burnout for the wellbeing coordinator. Such gaps and tensions reflect the frustrating experiences caregivers and practitioners encounter in the wider health and social care system in terms of inaccessible and unavailable support, as reported in other studies [3,35,36]. Although the WBC program, with its defined scope, cannot address all these challenges, sustained and adequate funding could facilitate enhancements, such as establishing a team of wellbeing coordinators to match the community demand and implementing a supervisory and community of practice structure around the role. This could help to not only meet demand but also support the wellbeing coordinators’ mental health and reduce the risk of burnout [16].

During implementation, the WBC program extended the number of appointments (more than six as initially designed) for families with highly complex needs, with two families having no less than 19 appointments. This suggests the dilemma: while program flexibility caters to complex family needs, it may limit program reach, potentially causing delays for eligible families to access the program given its limited capacity. To balance the service breadth and depth, flexible eligibility criteria and delivery of a program are required to meet the needs of families with varying levels of complexity. Implementing a tiered delivery approach may provide a viable solution [37]. Additionally, this highlights the important contribution of improvement science to understanding and evaluating Hubs in that while there is departure in fidelity from the original program logic during implementation, complex interventions require consistent iteration and adaptation to ever-evolving local contexts [38].

## Strengths and Limitations

To our knowledge, this is the first study that has evaluated the acceptability and feasibility of a program combining care navigation and social prescribing, in the context of an integrated health and social care Hub for families of young children experiencing adversity. A major strength of the research design was the inclusion of multiple stakeholder groups, including practitioners from various disciplines and organisations, caregivers who took part in the WBC program and those who did not. Additionally, a mixed-method design allowed a comprehensive evaluation by combining the objective process data and the subjective experiences of the stakeholders.

However, there were some limitations. The small sample size of caregiver participants and the use of only descriptive analysis warrant caution when interpreting the results. Additionally, the sample did not include caregivers who did not speak or read English, which may not be representative of families receiving services from the WBC program. The lack of children’s perspectives is another limitation, hindering a comprehensive assessment of the program’s impact on their wellbeing. This is understandable, as families experiencing adversity are often difficult to reach [39]. Additionally, some referral information was not collected due to confidentiality considerations, for example, the details of wellbeing plans developed for families. This resulted in an incomplete picture of the implementation of the WBC program, hindering a comprehensive understanding of the ways that families were asking to be supported and were provided with support. Moreover, given the overlaps between care navigation and social prescribing, it is difficult to distinguish the delivery of both with the referral data collected. Future research could conduct follow-up interviews with practitioners (especially wellbeing coordinators) to specifically understand the implementation process.

## Conclusion

Families experiencing adversity often have complex needs and face barriers to accessing health and social care. The WBC program was codesigned with and for these families. Our findings suggest that the WBC program is generally acceptable, feasible, and has the potential to alleviate caregivers’ loneliness and enhance their global health, connection to the community, and knowledge and confidence in managing their child and family health and wellbeing. One potential enhancement for the future of the WBC program is the recognition of adequate and ongoing funding to support the development, implementation and evaluation of the wellbeing coordinator role, ensuring that the WBC program is a pivotal and clear inclusion within a Hub and securing its sustainability as a key component. Other future improvements encompass establishing effective communication strategies for the WBC program to facilitate appropriate referrals by practitioners and enhance self-referral by caregivers, implementing support mechanisms for wellbeing coordinators to reduce the likelihood of burnout, and developing a tiered WBC program to meet the needs of the communities served by the Hub.

## Additional Files

The additional files for this article can be found as follows:

10.5334/ijic.8644.s1 Supplementary file 1. Semi-Structured Interview Schedule.

10.5334/ijic.8644.s2 Supplementary file 2. Supplemental Tables 1 and 2.
