# Supplementary material for: Acceptability, Feasibility, and Preliminary Effectiveness of a Wellbeing Coordination Program in an Integrated Health and Social Care Hub: A Mixed Methods Study
Source: Int J Integr Care. 2025 Feb 19;25(1):10. doi: 10.5334/ijic.8644 (PMC11843926; doi:10.5334/ijic.8644)
Supplement: Supplementary file 1. — Semi-Structured Interview Schedule. [file ijic-25-1-8644-s1.pdf]

Semi-Structured Interview Schedule

For caregivers who first got to know the Wellbeing Coordination Program:

Explain what the Wellbeing Coordination Program is: The Wellbeing Coordination Program is a new service at IPC Health, Wyndham Vale. The wellbeing coordinator is someone that works with your family to help you make links to other services and communities to help improve your family's overall wellbeing.

> *Note. Wellbeing can be explained as a state of **being comfortable, healthy or happy**. It includes various aspects such as **physical** well-being (e.g., being free from illness or discomfort), **mental and emotional** well-being (e.g., being able to cope with stress; feeling happy and satisfied with life most of the time), and **social** well-being (e.g., having good relationships within and beyond the family; not feeling isolated).*

1. Were you **aware** of this service?

   Prompts:

   - *If yes, how did you become aware of this?*
   - *If not, would you have found it helpful to know about the Wellbeing Coordination Program?*

2. Would you have found it **helpful** to **be referred to** the Wellbeing Coordination Program? *(Why, or why not?)*
3. Would you consider referring **yourself** to the Wellbeing Coordination Program? *(Why, or why not?)*

For caregivers who took part in the Wellbeing Coordination Program:

1. Can you tell me a bit about your experience with the Wellbeing Coordination Program?
   Prompts: Who referred you? How did that go for you? What helped? Anything not going well? Comfortable or not?
2. Did the wellbeing coordinator's support **make any changes/differences** in your child/family's wellbeing?
   Prompts: How? Or why not? Give an example?

For service providers who work for the Child and Family Hub (working with the wellbeing coordinator):

Tell me about your experience of the Wellbeing Coordination Program in the Child and Family Hub.

Prompts:

- *What was good about having a Wellbeing Coordination Program in the Child and Family Hub?*
- *What was the value of the Wellbeing Coordination Program to you as a practitioner or to your families? (examples)*
- *Did having the Wellbeing Coordination Program cause any harms? (Please explore why or why not?)*
- *How do think this program could be improved?*
