# Supplementary material for: Acceptability, Feasibility, and Preliminary Effectiveness of a Wellbeing Coordination Program in an Integrated Health and Social Care Hub: A Mixed Methods Study
Source: Int J Integr Care. 2025 Feb 19;25(1):10. doi: 10.5334/ijic.8644 (PMC11843926; doi:10.5334/ijic.8644)
Supplement: Supplementary file 2. — Supplemental Tables 1 and 2. [file ijic-25-1-8644-s2.pdf]

The supplemental file includes two tables outlining the key themes and representative quotes from the perspectives of caregivers and practitioners regarding their experiences with the WBC program.

Supplemental Table 1 *Caregivers' Attitudes Towards and/or Experiences of the WBC Program*

Theme	Code	Representative quotes
A 'valuable' program to know about	Awareness of the program	'I'm sure XX [paediatrician] has mentioned, look, asked if we need any help with finding anything, but […] because I don't need it, she's never elaborated. I think it's a great service though'. (Caregiver 8)
		'For me it's the capacity. […] Because I'm sitting here concentrating with you now and […] you've got my full attention, I can process it and go, "Hey, yes, that's really something I could use" and then I'm more likely to take it on. Rather than if I just glimpse at something or […] even for a couple of seconds […] it's not likely to stay in my brain because I've got way too much other stuff going on'. (C9)
	Willingness to be connected to the program	'If I need the support, I'll just refer myself for it. I don't think I need the support for now, in this situation, but I'm not sure what will happen in the future, and if I might need something like that for myself if I'm going through something, then why not? And now I'm aware that this service is available, and then I will just go'. (C10)
	Care navigation	'If you're dealing with mental health, you are probably dealing with other things, like relationships, so your social life and even financial. So being able to have someone that has all that expertise, and not just relating to one thing, would be extremely helpful to anyone that needed it'. (C3)
		'It's so complicated you go to one person for one thing, you go to someone else for something else and then somebody else for something else, and then trying to control them at the same time, is really tough'. (C9)
	Mental health and emotional support	'To be able to gain more understanding and just build myself. […] So if this service is available, it's for me to take part and learn how to cope and deal with other aspects of things in my life. […] Better knowledge on how to cope with certain things that are happening in my life that I wouldn't have normally known how to cope with. […] Anything to do with mental wellbeing, I guess'. (C17)

	Community connection	'Maybe some community groups and services, yeah. […] Like a [*sic*] parenting for people with Autism or like an Autism club or something for the kids […] Because then they understand while other people are just like oh my God [laughter]. […] I think [the groups] more just as like a, just an additional support like or, like, you know, ideas on how to parent them better or like, you know, even for the kids more of a social thing because they find it difficult to make friends'. (C14)
Reduced burden of linking to services	Learning about family and identifying needs	'She [the wellbeing coordinator] would ask about my father. She would ask about my children's father with, like, the court side and domestic violence side and stuff like that. We would talk about my son's diagnosis. Things that I've noticed about my daughter in preparation for when she gets assessed'. (C19)
	Care navigation	'She [the wellbeing coordinator] also had my back with the NDIS. There're a lot of problems with NDIS. And she's at the moment, trying to find a different service provider for me to go through. […] She's also on my side when I complain about NDIS. […] She's been a really big help with researching things like government grants and NDIS and she gives me everything'. (C19)
	Social prescribing	'My experience working with XX [the wellbeing coordinator] was really fantastic. She is really supportive in any I asked her. Because she's the one that linked me to the financial, to XX [the financial counsellor] and to the other one, to the family support'. (C20)
	Checking in, linking to services and following up	'She's [the wellbeing coordinator] like a person that just checks in. […] She'll ask me, "Is there anything you need? […] If there's something that she thinks, "Oh, okay, well, I might just flick an email to XX [paediatrician] about that"'. (C19)
		'She would ring me back and say, "How did it go with legal aid? Was she good? Was she not?" […] If I said, "No, she hasn't contacted me," she's like, "Okay, I'll email her again. I'll ring her again". (C19)
	Coordinating with other practitioners	'Especially the Support Worker when she came, 'she said, "Oh, XX's [the wellbeing coordinator] told me everything […] How can I help you with?" […] It's good because when you want to come to someone's place, you absolutely know a little bit of what's going on […] Because if you know what's going on, you'll know what to do, like where to start from or something like that I think'. (C20).
Emotional support brings hope	Trusting relationship	'It's good to talk to someone who listens. Like obviously, the paediatrician does that, too, but she has a timeframe, and she's there for the kids. Whereas XX [the wellbeing coordinator] is just like […] a pen

		pal'. (C19)<br><br>'She listens. She will ask questions, and then it's not just like a question where it's just a yes or no answer. It's a question where she invites conversation. […] I feel like I can open up, and I can talk to her. She'll give me a response back that's just not a yes or no answer. […] She's still super professional, but for me personally, it feels like two mums sitting in a room having a conversation about our kids. You need that'. (C19)
	Using lived experiences	'We get along great because she's a mum too. […] Obviously, we have that rapport. We can talk about it. […] One of her child [*sic*] being older than mine, so she, again, she can confirm to me that stuff is normal'. (C19)
	Emotional support brings hope	'She has patience, and the way she talked to me and the way she encouraged me. […] She's very supportive. It makes me feel like there's hope'. (C20)<br><br>"If I'm ever feeling overwhelmed or whatever, she'll reassure me that, "You're doing a great job. Don't forget you're doing this all on your own"'. (C19).
Positive impacts on wellbeing	Positive impact on parents and children	'I think the way forward. I don't stress the way I used to' (C20).<br><br>'I became way more calm, not walking on eggshells all the time, not worried about if I'm going to bump into their dad or anything like that'. (C19)<br><br>'They [children] started improving in school […] because of the environment they were in and I took them out of'. (C19)

Supplemental Table 2 *Practitioners' Experiences of the WBC Program*

Theme	Code	Representative quotes
Filling the 'cracks'	Care navigation<br><br>*(Filling the cracks between family's needs and available services/fragmented care system)*	'I guess having someone to touch base with, to have that directly so they could be linked in with community or programs, to be told what's available they can access just that kind of support. Because my client might tell me something, but they'll tell another service something completely different with what they're looking for at that time'. (Practitioner 16)<br><br>'Even if it's just getting the right information at the one place makes a big difference than just going from place to place'. (P23)
	Warm referrals<br><br>*(Filling the cracks between practitioners' intentions to meet the needs of families and their limited capacity)*	'It's just about giving people an option of that bit of extra support. If questions can't be answered immediately in other sessions, particularly around the NDIS, or, you know, referrals to ECEI [Early Childhood Early Intervention], which can take time. It's having a person there that's got that specific role to have a little bit more time to actually connect with the clients that the practitioners might not have'. (P9)<br><br>'If you were going to ask these questions and discuss these things and if it did end up being that the problem was too complex […] it was good to know that you had a fallback person that you could refer to, who's a bit more specialised and knowledgeable in that area'. (P17)
The wellbeing coordinator is the central connector	The centre/transit point of internal referrals	'That was the service I probably referred to the most, and I was in contact with the most because I feel like it's the role that's the most broad and covers lots of things. I'd often explain that service to clients when they brought up things like carer stress or funding or other services that they're looking for'. (P5)<br><br>'I just think that most of the referrals that I've seen have come through the wellbeing coordinator, so it seems that people are comfortable speaking with the wellbeing coordinator. […] They're opening up, speaking to the wellbeing coordinator, then there's referrals that come through. That to me seems to be working'. (P23)<br><br>'We did receive a few, I think maybe 2 or 3 referrals. So it was good to know that someone is supported with other areas as well, not just the legal aspect. So it was good to know that you could

		refer them back to the health and wellbeing coordinator for assistance. Having someone specific they can speak to, that's within the Hub practitioners, that was definitely a benefit'. (P19)
		'I think families found it helpful. I would check in and say, have you spoken to so and so about what we spoke about last time? They would say, "Yes. They're helping me with so and so". A lot of the time when I checked in, things were followed up, and they felt more in control of those situations they mentioned previously, so that was good'. (P5)
	Connecting practitioners to the Hub	'Whenever I've been here in the community […] like have a chat with her [the wellbeing coordinator] to see how things are going. I guess she is […] like a really accessible way for me to connect to the Hub. Because I know where she is, there is like time like set aside where I can pop in. Whereas I know with the others […] are very busy […] seeing patients or doing something else, and there isn't really that time'. (P22)
Need for a team of wellbeing coordinators	Concerns of overwhelming the wellbeing coordinator	'I also sometimes feel like I'm asking her to do too much. And so I'm like, "Do I have to pick and choose families, or do I just overwhelm her, and then she goes, I need help?". […] I think you need 3 or 4 [wellbeing coordinators]'. (P1)
		'I always wondered actually how busy XX [wellbeing coordinator] was. […] There's definitely times where I wasn't sure whether I should be referring or should be trying to manage the issues that were coming up myself'. (P17)
	A need to work in depth with families	'They [wellbeing coordinators] could perhaps look into deeper getting that assistance rather than someone, we can say, for example, "Here's a referral to this organisation", where they communicate with that organisation to get it in place, and that's why I think it would need to be just more than one person in that sort of role'. (P19)
		'The golden number of six sessions [number of sessions originally designed for the WBC program] has been […] truly thrown out the window. […] Six plus for most of them. […] So you touch base every now and then, they still want you to be in contact in case something happens'. (P9)
	The wellbeing coordinator felt her role isolating	'It's been quite an isolating role. You know, I was used to quite a large team, and now there's sort of only a team of one, and there was not a lot of interpersonal contact between the rest of the Hub outside

		of the lunchtime learning'. (P9)
Redefining the program scope	Narrow program scope fails to support high-needs families	'Well, when we were kind of told about the program, we assumed that it would be to do with help with some of our clients. But it wasn't until we'd started that we were like, Oh, okay, so being Child Protection, they can't be involved. No, Child FIRST [now transitioned to the Orange Door, a home-based program to prevent child maltreatment in Australia,] can't be involved. No, the social worker can't help them. […] I felt, well, that leaves out, you know, 80% of the clients. In that sense, that wasn't helpful'. (P15)
		'WBC was too limited as it did not include Child Protection'. (P3)
		'I think opening up the criteria would be really helpful. […] Sometimes we have complex clients, and they might have a bunch of services in health, but their services are inactive, or they're at closure or unable to have contact with the client. [….] For example, the majority of my clients can't be connected with family violence services because they're not looking to leave the relationship. So they're not given any service access unless they want to work on their parenting, which isn't always what they're looking for. So I think having more flexible criteria allows families to feel more connected, so my client could be linked in with other services rather than being told, because she's not ready to leave, she can't access anything right now'. (P16)
	Tensions between practitioners' expectations and WBC's practice	'Yeah, so I understand Child Protection, okay, they can go to Child FIRST, they can do this, they can do that. But I'm like, "Why couldn't you [the wellbeing coordinator] do something in the meantime?"' (P15)
	The role is hard to understand	'It's not as clear as saying like I'm a doctor, I'm a speech pathologist with very clear [descriptions]. Like people know what you do'. (P20)
	Confusing the wellbeing coordinator role with other social worker roles	'It was a bit confusing because I thought, "Great, social worker." Now, I have other services involved doing social work stuff that she [the wellbeing coordinator] wouldn't do that'. (P15)
		'I think the scope of things that she assisted with maybe was more narrow [*sic*] than some social workers that I've seen'. (P20)
	The need to understand the	'I think I need that to be defined better, and I need to understand it more because I think there could be

	role of the wellbeing coordinator	more opportunities for financial counselling and wellbeing to work together'. (P18)<br><br>'Maybe it could have been like clearer about what … she could and couldn't do. Yeah. Rather than it just people generally being like, oh, yeah, I know she exists, and that she helps connect people with the services'. (P20)<br><br>'Maybe articulating what sort of things the wellbeing coordinator can do a little bit more clearly because it might actually help family self-refer as well. […] So, the wellbeing coordinator can help you with XYZ?' (P1)
